# Large erythema migrans lesion in Lyme disease

**DOI:** 10.1002/jgf2.297

**Published:** 2020-01-30

**Authors:** Yuki Takeuchi

**Affiliations:** ^1^ Teine Family Medicine Clinic Sapporo Japan

## Abstract

This study reports a case of Lyme disease with a large erythema migrans in Hokkaido. This image aims at encouraging physicians to consider Lyme disease after tick bites, especially in endemic areas.

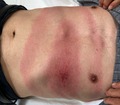

An 80‐year‐old Japanese man presented to a clinic in Hokkaido with a painful, itchy abdominal erythema that had been expanding for 5 days. He reported having been bitten by a tick on his abdomen 10 days prior. He denied having fever, headache, fatigue, arthralgias, or myalgias. He was alert and oriented. On examination, the temperature was 36.1°C, the pulse 86 beats per minute, the blood pressure 140/75 mm Hg, and the oxygen saturation 96%. Examination revealed a nontender, oval (32 × 66 cm) homogeneous erythematous lesion at the tick‐bite site, consistent with erythema migrans (Figure 1). On the basis of the patient's history and examination, Lyme disease was suspected. The Borrelia antibody test (Western blotting) showed an increased serum anti‐Borrelia IgM level. Furthermore, skin biopsy revealed Borrelia garinii DNA by polymerase chain reaction. These findings confirmed the diagnosis of Lyme disease. Doxycycline was prescribed for 14 days at the first visit. His erythema migrans disappeared at day 7 of treatment without other symptoms (Figure 2).

**Figure 1:**
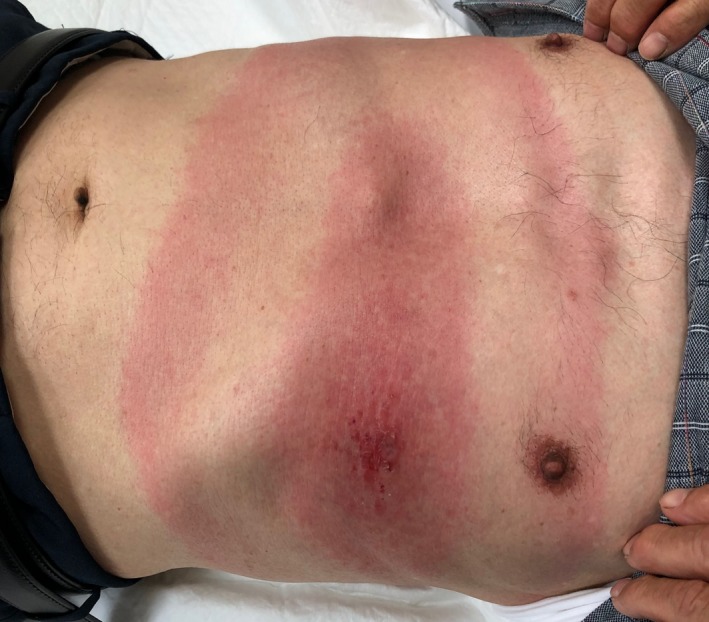
Erythema migrans on the abdomen

**Figure 2:**
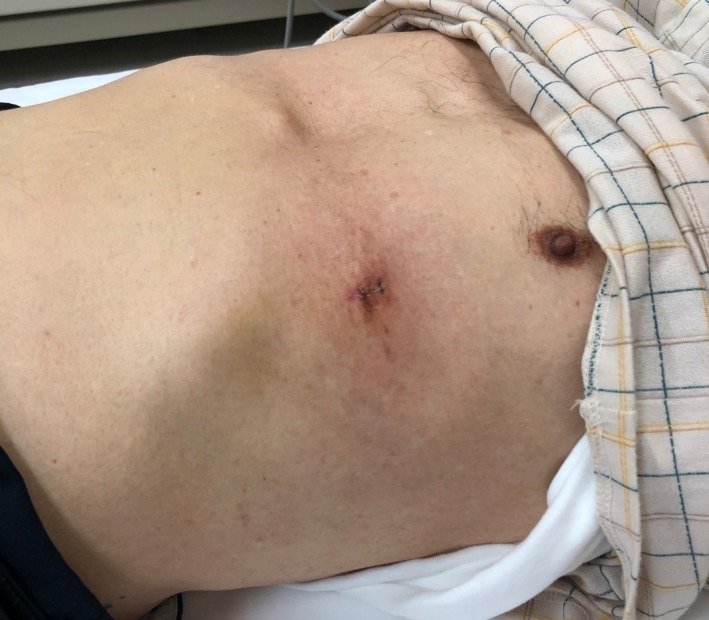
Erythema migrans disappeared at day 7 of treatment. (There are two skin biopsy scars on the left flank)

Lyme disease is a tick‐borne disease caused by Borrelia infection that can cause several complications (carditis, atrioventricular block, chronic arthritis, peripheral neuropathy, or encephalopathy). Physicians in endemic areas should carefully examine patients after tick bites because many cases of Lyme disease may be undiagnosed. The prevalence of *Borrelia* infection in *Ixodes persulcatus* ticks was 34.5% in Hokkaido, Japan,^1^ similar to that in Finland.^2^ Although the incidence of Lyme disease is more than 50 cases per 100 000 population per year in Finland, only five cases were reported in 2018 in Hokkaido (0.1 cases per 100 000 population per year).^3^, ^4^

Early diagnosis is important to prevent complications. The Infectious Diseases Society of America guidelines suggest that the clinical manifestation of erythema migrans is sufficient to make a diagnosis of Lyme disease in the absence of laboratory confirmation.^5^ Erythema migrans is characterized as an area of round, flat or slightly raised, erythema that expands in diameter over days to weeks after tick bites. The median diameter is 16 cm (range, 5‐70 cm).^6^ Erythema migrans, in this case, was large. In actual clinical situations, distinguishing between erythema migrans and tick‐bite hypersensitivity could be challenging. In contrast to erythema migrans, the largest diameter of hypersensitivity reaction is <5 cm, and it usually resolves within 24‐48 hours. Thus, observation for 1‐2 days without antibiotics is useful to make the correct diagnosis.^5^

## ACKNOWLEDGMENT

None.

## CONFLICT OF INTEREST

The authors have stated explicitly that there are no conflicts of interest in connection with this article.
